# The Psychosocial Dimension of Electrical Burns Related to Work Accidents—A Phenomenological Study on the Experiences of Patients Fighting for Their Lives in Intensive Care in Turkey

**DOI:** 10.3390/healthcare14040542

**Published:** 2026-02-22

**Authors:** Serpil Çelik Durmuş, Sevda Uzun

**Affiliations:** 1 Department of Nursing Management, Faculty of Health Sciences, Kırıkkale University, 71100 Kırıkkale, Turkey; 2 Department of Psychiatric Nursing, Faculty of Health Sciences, Kırşehir Ahi Evran University, 40100 Kırşehir, Turkey; sevdauzun50@gmail.com

**Keywords:** Turkey, electrical burns, psychosocial difficulties, phenomenological study

## Abstract

**Highlights:**

- This phenomenological study explored the psychosocial difficulties experienced by individuals who survived electrical burn injuries.
- Participants reported intense negative emotions, including depression, helplessness, and hopelessness, following the injury.
- Social appearance anxiety and impaired body image were among the most significant psychosocial challenges.
- Survivors described difficulties in coping during both the acute hospital phase and the post-treatment adaptation process.
- Many participants emphasized the need for psychological support and reported challenges in managing long-term emotional and social consequences.

**What are the main findings?**

- According to the results of the research, it is understood that individuals experienced negative emotions such as depression, helplessness and hopelessness as a result of electrical burns and could not cope with the psychosocial difficulties experienced and received psychological support. It was determined that social appearance anxiety due to deterioration in body image was very important in individuals.

**What are the implications of the main findings?**

- This study may contribute to the identification of psychosocial difficulties experienced by individuals who have experienced electrical burns.

**Abstract:**

**Background:** Electrical injuries occur when an electric current comes into contact with or passes through the body. Electrical injuries can result from contact with faulty electrical appliances and machinery or from contact with open household wiring or electrical power lines. **Aim:** The aim of this study was to evaluate the psychosocial difficulties experienced by individuals who suffered electrical burns due to work accidents, using a phenomenological approach. **Study Design:** This phenomenological study was conducted with semi-structured in-depth interviews with 15 electrical burn survivors living in different regions of Turkey via the WhatsApp mobile application. The snowball sampling method was used to reach the sample group. Interviews continued until data saturation was achieved. All interviews were audio recorded and then transcribed. The data were analyzed using Colaizzi’s phenomenological analysis method. The study was conducted and reported according to the COREQ checklist. **Results:** In the analysis of the data, two categories and five themes were identified: the effects of electrical burn at the time of occurrence and during the hospital process (psychological, social and physical), and adaptation to life after electrical burn treatment (emotions experienced, difficulties experienced and coping). **Conclusions:** This study revealed the life experiences, psychosocial difficulties and coping experiences of individuals with electrical burns. According to the results of the research, it is understood that individuals experienced negative emotions such as depression, helplessness and hopelessness as a result of electrical burns, could not cope with the psychosocial difficulties experienced and received psychological support. It was determined that social appearance anxiety due to deterioration in body image was very important in individuals.

## 1. Introduction

Electrical injuries occur when an electric current comes into contact with or passes through the body. Electrical injuries can result from contact with faulty electrical appliances and machinery or from contact with open household wiring or electrical power lines. Electrical injuries can range from minor skin burns to life-threatening internal organ damage. An electric current can cause localized damage to the skin or muscles or affect other organs such as the heart. Skin burns may occur at contact sites, but most do not result in serious injury. The severity of injury depends on the voltage, type of current and duration of contact [1]. The electric current is either direct or alternating. An alternating current prevents people from leaving the electrical source and is more dangerous, as it can cause muscle contractions. In addition, longer exposure to the electric source leads to more serious injuries. Electrical injuries can affect the skin (burns), muscles (convulsions or tissue damage), heart (arrhythmia or cardiac arrest), brain (seizures or loss of consciousness), eyes (abnormal vision) and nerves (abnormal sensation, difficulty speaking, swallowing, weakness, paralysis) [1,2].

The devastating effects of electric shock can be seen as compartment syndrome, renal failure, organ loss, burn injury, trauma findings, cardiac arrhythmia, respiratory arrest and death. In low-voltage electrical injuries, burns are usually more superficial and muscle destruction is rare. Life-threatening cardiac arrhythmias, especially ventricular fibrillation, respiratory arrest and trauma, can be seen in both types [3]. Electrical injuries account for approximately 0.04–5% of burn unit admissions in developed countries and 27% in developing countries. Classification of electrical injuries is typically categorized as low-voltage (1000 volts) and is also according to whether the electric current flows directly through the body and whether thermal injury is caused by an electric shock. Electrical injuries in the adult population mainly affect men, are mostly work-related and are the fourth leading cause of work-related traumatic deaths. Both morbidity and mortality are relatively high in electrical injuries and have both physical and psychological short- and long-term sequelae [4,5].

Disorientation, confusion, delirium, transient psychosis, depression and anxiety, stress and sleep disturbances are frequently observed during acute treatment of patients with electrical burns during hospitalization. The altered state of consciousness may be transient, waxing and waning, and may last for weeks in the case of major burns. During recovery, symptoms of acute and post-traumatic stress disorder can be seen in connection with the sudden revival of memories of the injury in the mind [6].

Burns can lead to many different psychosocial problems. Depression and agitation due to excessive pain and related anxiety and feelings of powerlessness may occur. It has been reported that patients experiencing high levels of pain have more adaptation problems after discharge, have a higher risk of psychiatric problems, and accordingly, the wound healing rate is slow. In addition, high levels of stress, anxiety and traumatic disorder may decrease pain tolerance. Itching during wound healing can affect the stress level, and in this sense, the rehabilitation process is negatively affected [6,7,8]. Electrical burns are significantly more common in males. It is thought that such burns are more frequently seen in males because males are generally more involved with electrical and electronic devices [9]. Studies have shown that 13–23% of patients with electrical burns have depression, 13-45% have post-traumatic stress disorder, and there are also some neuropsychological problems due to electrical injuries. Socially, there may be difficulties in social interaction and sexual life and initially low quality of life. Psychosocial problems that occur after recovery include body image changes, coping with loss and adaptation problems [8,10].

The diagnosis and treatment of patients who survive electric shock is quite complex. Electrical injuries negatively affect individuals psychosocially. In this context, this phenomenological study aims to shed light on the life experiences of survivors by comprehensively describing the problems they experience during and after the treatment process. Although there is a wealth of terminological information in the literature regarding electrical burns related to occupational accidents, there are only a limited number of phenomenological studies addressing the psychosocial dimension of electrical burns. This study was conducted to fill a gap in the literature, as there are very few qualitative studies addressing electrical burns psychosocially.

## 2. Materials and Methods

### 2.1. Study Design

Throughout this study, the authors followed the Consolidated Criteria for Reporting Qualitative Research (COREQ) and reported it accordingly [11].

This study was conducted in November 2023, using an inductive qualitative design. In-depth individual interviews were conducted with 15 individuals living in different regions of Turkey who experienced electrical burns due to work accidents, via a telephone conversation using the WhatsApp mobile application.

### 2.2. Research Team and Reflexivity

The researchers work as active faculty members at the nursing school. One of the researchers holds a doctorate in psychiatric nursing and the other in nursing administration. Both researchers have experience working as clinical nurses in a hospital and have received training in qualitative research methods.

### 2.3. Working Group

The survey sample was created using the snowball sampling method, and individuals living in different regions of our country were interviewed via telephone conversation using the WhatsApp mobile application. Interviews continued until the data were repeated and were terminated when data saturation was reached by interviewing 15 men.

The inclusion criteria were as follows: (a)suffering an electrical burn due to a workplace accident, (b) having been hospitalized for burns, (c) being open to communication, and (d) agreeing to participate in the study. The exclusion criteria were as follows: (a) having a speech, language or hearing impairment that prevented communication and (b) not agreeing to participate in the study.

### 2.4. Participants

The mean age of the participants in the study was 37.46 ± 8.10 years; all of them were male and eight of them were married. In addition, seven of the participants were electrical technicians. In addition, eight of the participants had trunk burns, three had head/face burns, and four had extremity burns. The burn depth was of the third degree in eight of the participants and the total body surface area affected was over forty percent foreight of the participants.

### 2.5. Data Collection

A semi-structured interview form was prepared by the researcher, based on the related literature review. The form consists of two parts. In the first part, there are 6 questions about the age, gender, occupation, marital status, and place of residence of the individuals who have experienced electrical burns. The second part consists of a form consisting of six basic open-ended questions to be used in a semi-structured interview. The questions in the semi-structured interview form were individually used in interviews with people who had experienced burns via WhatsApp [12]. In the interviews, individuals who had experienced electrical burns were asked to describe the problems they experienced, their experiences with struggle, and the questions “Can you explain your answer a little more?” and “What do you mean by this?” All interviews were conducted by the researcher. The interviews were recorded using a Sony voice recorder and transcribed verbatim by the researcher. After all interviews were completed, the study data were transcribed for analysis. The questions in the semi-structured interview form are as follows:

1. How do you feel when you remember the moment you received an electric burn?
2. What have you experienced during the electric burn treatment process? What are your experiences?
3. What were the psychological, social and physical effects of the electric burn incident on you? What are your experiences?
4. How was your adaptation to life after electric burn treatment?Can you tell us about your experiences in the process? Do you think you coped effectively?
5. What are the effects of electrical burns on you and your family? What are your experiences?
6. How do you feel now? Are there any difficulties you are experiencing?

### 2.6. Trustworthiness and Rigor

The researcher also conducted a peer review to strengthen the reliability of the study. This process involved structured discussions with colleagues who were familiar with qualitative research methods and provided an opportunity for critical thinking about the findings and analytical process. Peer review helped to identify potential biases and ensured that the study’s analysis was reliable and well-supported by the data. To increase the rigor of the research, the researcher kept a reflective journal throughout the research process. This journal documented personal reflections, potential biases, and assumptions that arose during data collection and analysis. Through reflection, the researcher aimed to remain aware of how individual perspectives could influence the study and to consciously reduce these biases [13]. The journal also allowed the researcher to track changes in data interpretation throughout the study, providing a more nuanced understanding of participants’ evolving experiences. Additionally, an audit trail was maintained to document each step of the data analysis process; this ensured transparency and facilitated the replicability of the study. This audit trail strengthened the rigor and accountability of the study by containing detailed records of decisions made during the coding process, thematic development, and analysis [14]. By employing these strategies for peer review, reflective journaling, and audit trail documentation, the study maintained reliability and rigor, ensuring that the findings were credible and grounded in participants’ actual experiences.

### 2.7. Data Analysis

In the analysis of the qualitative data obtained from the interviews, the 7-step analysis method developed by Colaizzi (1978) for phenomenological studies was used [15]. In this context, the interview texts were first read independently and repeatedly by two researchers. Thus, they tried to understand what was explained in the data. Important statements in the interview texts were selected, reorganized and expressed in general terms. Then, the data that were explained in the statements were identified and analyzed. The researchers formulated and validated the meanings by discussing them until they reached a consensus. The researchers then identified and organized the themes into main and sub-themes. The themes and sub-themes of the study were developed through clear articulation. In addition, participants’ statements were included so that the reader could verify the interpretation and analysis of the data [16,17].

### 2.8. Ethical Aspects of the Research

This study was approved by the X University Scientific Research and Publication Ethics Committee. Informed consent was obtained from the participants before starting the interview. Recordings and transcripts were stored on a password-protected device. The study was conducted in accordance with the Declaration of Helsinki and the ethical standards of the National Research Committee.

## 3. Results

Demographic characteristics of the individuals who participated in the study are presented in Table 1.

As a result of the analysis of the data obtained from the semi-structured interviews, themes, sub-themes and codes were identified (Table 2).

The effects of electrical burns at the time of occurrence and during the hospital process.

### 3.1. Subtheme 1: Physical Effects

According to the data obtained from the interviews, individuals who experienced electrical burns stated that they experienced physical conditions such as general body pain, difficulty breathing, pain in the graft areas, burns on all limbs, limitation of movement, frequent surgical operations, inability to fulfill self-care and hypothermia/chills in hospital clinics or intensive care units.

*“There was no concept of time during the treatment process, it was like a movie that lasted intermittently while I was in intensive care. I didn’t feel much pain, but what I remember was that I couldn’t breathe, and when I removed the hose attached to my throat, I started to breathe a little bit and then I relaxed, and then when I moved to the other intensive care unit, I came to my senses a little bit, and the pain of my cheek and the pain of the graft areas of the pieces of skin taken from my body to the burn areas was too much, I was constantly in and out of surgery, but the worst thing was that unfortunately I didn’t know that my face was burned, it was a very difficult process for me…” (P4).*

### 3.2. Subtheme 2: Psychological Effects

Individuals who suffered electrical burns experienced mental problems such as anxiety, depression, pessimism, hopelessness, helplessness, fear of loss (organ, life), future anxiety, uncertainty, and perception of deterioration in body image in intensive care or clinics.

*“It was really a difficult time for me, sometimes I talk to myself and I wish I hadn’t gotten into that nightmare, that fireball, but sometimes you don’t know, but in real terms, this burn affected me a lot both physically, mentally and socially, but I can say that the mental destruction is the most because it is the moment when it really ends you at the point of hope.” (P1).*

### 3.3. Subtheme 3: Social Impacts

Individuals who experienced electrical burns stated that they experienced social anxiety, decreased self-confidence, thinking that people at their job would not like them and thinking about how they would live in society due to facial burns in intensive care or clinics.

*“There is a saying in our country that I can’t tell you, you have to live it, God forbid that no one should experience what I went through, what we went through is not something that can be described neither with language nor with a pen, it is very difficult to experience hell in this world, and I really experienced it to the fullest, while I was in intensive care, I always thought about what will happen to the burns on my face, what will I do, how will I walk around in public, aside from my pain, one of the things that worried me the most was the burns on my face.” (P12).*

### 3.4. Adaptation to Life After Electrical Burn Treatment

#### 3.4.1. Subtheme 1: Experienced Emotions

Individuals also reported experiencing negative emotions such as anxiety, depression, helplessness, burnout and hopelessness after burns.

*“I am having an adaptation problem right now, I am not used to this situation, I am meeting new people, I mean, I have serious injuries on my body, especially a big burn on my face. Burns cause depression, helplessness and hopelessness in people, unfortunately I don’t want to go out in public, when I go out in public, I feel really bad when I am exposed to everyone’s strange looks because of the burn on my face.” (P13).*

#### 3.4.2. Subtheme 2: Difficulties Experienced

Individuals who experienced electrical burns stated that they were exposed to strange looks from people, curious questions, emotional crisis, suicidal thoughts, feeling that their spouse did not like them, wanting to get away from society, post-traumatic stress disorder, sleep problems and mobility problems.

*“In the early days, on very difficult days, I was going out in the evenings, I couldn’t go out during the day, I couldn’t go out during the day, I tried to go out wearing a hat, if I had to go out during the day, if I had to go out during the day, the reactions of people’s strange looks when I went out caused me great distress, but there were times when I had to go out, after a while I tried to get used to it, and then what happened? In the early days, I used to tell people, I used to answer their curious questions, now I don’t tell them, sometimes I say something happened and I pass it off, frankly, I don’t think I can cope very effectively, I don’t think our people are very curious, I think that making a person tell about the event that a person has experienced can be experienced over and over again every time, those old days can be prepared and I think it creates great trauma in people.” (P9).*

#### 3.4.3. Subtheme 3: Coping

It was determined that individuals tried to cope with adaptation to life at the end of the burn treatment process by using psychological support, supporting hope, wearing glasses or hats when going out, continuing to struggle, getting the support of their spouse and family, going out in the evenings, engaging in favorite hobbies, breathing and relaxation exercises, and trying to forget those intensive care days and embracing spirituality.

*“I am trying to cope with the troubles caused by the burn, and my biggest supporter is my husband here, thank God I have my hands and feet in place, I need to hold on to life for my husband and children, I need to hold on to life for them, I love them very much because, as I said, I have taken up different hobbies to keep myself alive, but my biggest supporter is my family.” (P7).*

## 4. Discussion

The aim of this study was to evaluate the psychosocial difficulties experienced by individuals with electrical burns, using a phenomenological approach. The psychosocial dimension of electrical burn is addressed in two categories.

### 4.1. The Effects of Electrical Burns at the Time of Occurrence and During Hospitalization

In the study, individuals experienced many physical, psychological and social problems at the time of electrical burn and during the hospitalization process. It was determined that the problems experienced affected the individuals very negatively. Electrical injuries occur when the human body comes into contact with an electric arc, due to electricity passing through the human body. Electrical injuries are rare but potentially devastating and account for approximately 0.04% to 5% of admissions to burn units in developed countries and up to 27% in developing countries [18]. Although electrical burns are less common than other types of burns, they have high mortality and morbidity rates. In particular, a high-voltage electric current can cause arrhythmias or respiratory muscle paralysis, leading to sudden death, as well as morbidity and long-term dysfunctions and tissue damage as a result of severe burns and multiple system involvement. These include peripheral neuropathy and cataracts [19].

The passage of electrical energy through the human body triggers some very complex reactions in the tissues and organs of the body. These reactions affect the anatomical, histological and biochemical structure of the organism on the one hand and cause a series of electro trauma on the other [20]. The external signs of electrical burns may be deceptively mild. There is usually extensive underlying soft tissue damage. Nerves, vessels and muscles are more susceptible to electrothermal burns than bone and skin. Severe myonecrosis can produce myoglobinemia and renal failure. Tissue destruction is usually severe and progressive, due to vascular injury caused by the electric current and prolonged thromboxane production. Involvement of the abdominal wall can cause severe destruction of the wall itself and even the internal organs. The gallbladder, liver, pancreas, intestine and colon can all be involved. In electric shocks, the current forms a closed circuit and can cause internal organ damage on wet ground, depending on the length of the electric shock. Limb compartment syndrome is common and early decompressive fasciotomy is considered in this case [18,21]. In the study, it was determined that burns were physically very effective in individuals and surgical procedures were frequently performed in individuals, especially trying to close the defects by applying grafts. It can be said that this situation affects individuals very negatively.

Many systems in the body such as cardiovascular, respiratory and nervous systems are affected as a result of electric shock. It has been reported that a secondary respiratory arrest may develop with contractions of respiratory muscles as a result of an electric current passing directly or indirectly through the chest. In a previous study conducted in Turkey, it was reported that the most common cause of death was associated with respiratory arrest and that general body trauma and burns were important factors in increasing mortality [10,22].

Burns have a significant negative impact on individuals physically, socially, and psychologically. Burns can cause temporary or permanent deterioration in the psychological state of the patient, due to severe pain during or after the injury, changes in appetite, changes in appearance, restriction of physical activities, and a sudden and long-term treatment process. The patient’s physical, social, emotional and economic structure and activities of daily living are negatively affected. Accordingly, it is more likely that the patient’s quality of life will decrease, they will suffer more psychological pain and show many symptoms of psychological health disorders [23,24]. It can be said that the individuals in the study were negatively affected psychosocially.

### 4.2. Adaptation to Life and Coping After Electrical Burn Treatment

As a result of the study, the adaptation of individuals to life after electric burn treatment was quite difficult and their coping experiences were quite painful. Electrical burns have complex, time-consuming, unpredictable outcomes, challenging care and treatment from hospitalization to discharge and even after discharge [19]. Psychological distress is among the most common and debilitating complications after burn injury, due to painful dressings, physical therapy procedures, changes in body image and structure, social discomfort, external stigma and disturbing reactions [24]. Difficulties in adapting to a changed appearance can cause individuals to turn away from mirrors, anxiety, embarrassment, social avoidance and isolation, and affect quality of life [25]. According to the study conducted by Oaie et al. the relationship between facial burns and the severity of depression was found to be high, which caused facial disfigurement to be among the risk factors for post-burn depression. In the same study, it was found to be statistically significant that patients with deep burns had anxiety, depression and low self-esteem [26].

As in our study, the psychological and social effects of electrical burns are quite significant. Fear of being evaluated negatively for one’s appearance is also associated with high social anxiety and/or social impairment [27]. Social appearance anxiety, which is defined as a type of social anxiety, is defined as the anxiety and tension that individuals experience about the evaluation of their physical appearance by others. In other words, it is the emotional reaction that people show against the evaluation of their physical appearance, as well as the characteristics of body perception and body image, such as skin color, face shape (nose, eye distance, smile) and muscle structure by other people [28,29,30]. This shows that our study is consistent with the literature.

Ayhan et al. conducted a study with the participation of 10 patients in a burn center outpatient clinic and concluded that the social appearance anxiety scores of the patients were moderate. In the same study, it was observed that being single, having a high level of education, having burns on the face, head or neck, having amputation due to burns and exceeding one week after the injury significantly increased social appearance anxiety [31]. In this study, individuals had problems with adapting to life after burn treatment and tried to cope. It was determined that some individuals had difficulty in coping. This situation can be said to be important in terms of showing that electrical burns are an issue that should be addressed in the psychosocial dimension.

## 5. Conclusions

This study revealed the life experiences, psychosocial difficulties and coping experiences of individuals with electrical burns. It was determined that individuals went through a very painful process after electrical burns. According to our research results, individuals experience negative emotions such as depression, helplessness, loneliness and hopelessness as a result of burns. Individuals’ mental health and family and social relationships are also negatively affected after burns. Individuals distanced themselves from society and isolated themselves as a result of the internalized stigma they experienced. It is also understood that some individuals could not cope with the psychosocial difficulties they felt and received psychological support. It was determined that social appearance anxiety due to a deterioration in body image was very important in individuals. It is recommended to develop institutional programs to reduce psychological distress due to social appearance anxiety in individuals.

## 6. Limitations

One of the limitations of the study is that all participants were selected from different regions of Turkey. The results depend on the participants and the setting in which the research was conducted. The small group of participants is not representative of the country’s population of individuals with electrical burn survivors. The interviews with the referees themselves have been quite exhausting and distressing for the individuals involved in terms of recalling that traumatic experience. In this context, the data has not been sent to the members for re-verification. Since it was not possible to reach individuals throughout the country in person, the interviews were conducted via WhatsApp in the form of telephone conversations. This is also one of the limitations of the study. The fact that all participants were male can also be considered to be one of the limitations of the study.

## Figures and Tables

**Table 1:** Characteristics of participants.

Participant Number	Age	Gender	Marital Status	Profession	Place of Residence	Economic Situation	TWWA (Total Body Surface Area) (%)	Anatomical Burn Sites	Burn Depth
K1	29	Male	Single	Electricaltechnician	Province	Incomebalancedtoexpenditure	45	Body	3rddegree
K2	46	Male	Married	Constructionworker	District	Incomelessthanexpenditure	18	Head/face	2nd degree
K3	28	Male	Single	Constructionworker	Province	Incomelessthanexpenditure	27	Thigh—leg Foot	2nd degree
K4	29	Male	Single	Electricaltechnician	Province	Incomeandexpenditurebalanced	45	Body	3rddegree
K5	33	Male	Married	Tradesmen	Province	Incomelessthanexpenditure	27	Thigh—leg Foot	2nd degree
K6	52	Male	Married	Electricaltechnician	District	Incomelessthanexpenditure	45	Body	3rddegree
K7	27	Male	Single	Constructionworker	Province	Incomelessthanexpenditure	18	Head/face	2nd degree
K8	46	Male	Married	Constructionworker	District	Incomemorethanexpenditure	45	Body	3rddegree
K9	31	Male	Single	Electricaltechnician	Province	Incomelessthanexpenditure	27	Thigh—leg, Foot	2nd degree
K10	41	Male	Married	Electricaltechnician	District	Incomelessthanexpenditure	45	Body	3rddegree
K11	36	Male	Married	Electricaltechnician	District	Incomebalancedtoexpenditure	18	Head/face	2nd degree
K12	40	Male	Single	Constructionworker	Province	Incomelessthanexpenditure	27	Upper extremities, Head, face	3rddegree
K13	36	Male	Single	Electricaltechnician	District	Incomelessthanexpenditure	45	Body	3rddegree
K14	39	Male	Married	Constructionworker	Province	Incomebalancedtoexpenditure	45	Body	3rddegree
K15	49	Male	Married	Electricaltechnician	District	Incomebalancedtoexpenditure	45	Body	3rddegree

**Table 2:** Psychosocial dimension of electrical burn.

Themes	Sub Themes	Codes
**1. The effects of electrical burn at the time of occurrence and in the hospital process**	A. Physical effects	A1. General body pain. A2. Difficulty breathing. A3. Intensepaininthegraftedareas. A4. Burns to all limbs. A5. Limitation of movement. A6. Frequentsurgicaloperations. A7. Inability to fulfill self-care. A8. Hypothermia/chills in intensive care unit.
	B. Psychological Effects	B1. Anxiety. B2. Depression. B3. Pessimism. B4. Hopelessness. B5. Despair. B6. Fearofloss. B7. Future anxiety. B8. Uncertainty. B9. Perceptionofdeteriorationinbodyimage. B10. Fear of death. B11. Grief. B12. Fear of internal damage. B13. Thinking that the spouse will not like him/her.
	C. Social Impacts	C1. Social anxiety due to facial burns. C2. Decreased self-confidence. C3. Thinking about how to live/circulate in society. C4. Going out in the evenings but not during the day.
**2. Adaptation to life and coping after electric burn treatment**	A. Emotion sex perienced	A1. Anxiety. A2. Depression. A3. Burnout. A4. Hopelessness. A5. Despair. A6. Future anxiety. A7. Suicidalideation. A8. Unhappiness. A9. Internalizedstigma. A10. Loneliness. A11. Low self-esteem. A12. Mourning for the burning face. A13. Social isolation.
	B. Challenges faced	B1.Gettingstrangelooks from people. B2. Being exposed to people’s curious questions. B3.Experiencinganemotionalcrisis. B4. Thinking about suicide. B5. Thinking that his/her spouse does not like him/her. B6.Thedesiretowithdrawfromsociety. B7. Post-traumatic stress disorder. B8. Sleep problems. B9. Movement problems. B10. Resenting mirrors. B11. Problemsinsexuallife.
	C. Coping	C1. Receivingpsychologicalsupport. C2. Supporting hope. C3. Wearingglassesorahatwhengoingout. C4. Continue the struggle. C5. Gettingsupportfromspouseandfamily. C6. Going out in the evening. C7. Engaging in favorite hobbies. C8. Breathing and relaxation exercises. C9. Tryingtoforgetthosedaysofintensive care. C10. Embracingspirituality. C11. Crying. C12. Praying.

## Data Availability

The data that support the findings of this study are available from the corresponding author upon reasonable request.

## Abbreviations

The following abbreviations are used in this manuscript:

P	Participant
